# Nature-based and technology-assisted exercise for cognitive and mobility outcomes in older adults: a systematic review of randomized trials

**DOI:** 10.1186/s12877-026-06978-x

**Published:** 2026-01-31

**Authors:** Guhua Jia, Chun-Hsien Su

**Affiliations:** 1 Sports Teaching Department, Shanghai University of Medicine & Health Sciences, Shanghai, 201318 China; 2 Department of Physical Education, Putian University, Putian, 351100 China; 3 Department of Exercise and Health Promotion, Chinese Culture University, Taipei, 111396 Taiwan

**Keywords:** Older adults, Mild cognitive impairment, Executive function, Dual-task gait, Nature-based exercise, Virtual reality

## Abstract

**Background:**

Population aging and rising rates of mild cognitive impairment and mood symptoms have intensified interest in low-cost strategies to preserve brain health and independence. Physical activity is an established protective factor, but adherence to exercise programs remains suboptimal. Emerging work suggests that the exercise environment, including natural settings and immersive or interactive formats, may confer benefits beyond conventional indoor training.

**Methods:**

We systematically searched major electronic databases for randomized trials in older adults that compared nature-based or outdoor exercise, or immersive and interactive modalities (virtual reality or exergaming), with broadly dose-matched indoor or usual-exercise control conditions. Eligible studies enrolled older participants or reported stratified results for those aged at least 60 years or with mild cognitive impairment. Primary outcomes were brain-related measures spanning executive function and global cognition, mood and perceived restoration, dual-task gait, autonomic and endocrine stress markers, and indices of brain activation or connectivity. Risk of bias was assessed with the Cochrane risk of bias 2 tool for randomized trials (RoB 2), and findings were synthesized narratively.

**Results:**

Twelve randomized trials met the inclusion criteria. Technology-assisted interventions that embedded cognitive demands in movement tasks produced small-to-moderate improvements in executive function, working memory, and dual-task gait, particularly in cognitively vulnerable older adults, compared with conventional exercise. Nature-based or outdoor sessions consistently enhanced affective valence, enjoyment, and perceived restoration relative to indoor or urban comparators, whereas effects on mobility, heart-rate variability, cortisol, and other physiological markers were smaller and heterogeneous. Functional near-infrared spectroscopy data suggested more efficient prefrontal recruitment during walking after interactive or enriched exercise. Overall risk of bias was low to some concerns, with small samples, short follow-up, and inconsistent exposure reporting limiting certainty.

**Conclusions:**

The context in which older adults exercise appears to be a modifiable component of brain-focused training. Immersive and interactive formats may enhance executive control and dual-task gait and mobility, while nature-based sessions reliably support affective and restorative responses that may facilitate adherence. Future trials should more rigorously match physical activity dose, standardize reporting of environmental and immersion characteristics, extend follow-up, and include core outcome batteries across cognitive, affective, physiological, and neuroimaging domains to identify context features that yield clinically meaningful benefits.

**Supplementary Information:**

The online version contains supplementary material available at 10.1186/s12877-026-06978-x.

## Introduction

### Background and rationale

 Aging populations and the increasing burden of mild cognitive impairment and mood-related symptoms pose a public health challenge, particularly among the elderly who need to preserve brain resilience and functional independence [1–3]. Engaging in physical activity is considered one of the simplest and most cost-effective approaches for ensuring the cognitive and emotional well-being of the human population across the life course, although training adherence remains suboptimal [2, 3]. Recent works have thus re-directed attention away from the mode and amount of exercise to the training environment, i.e., where and how to do physical activity [1–3].

Blue and green environments offer multiple sensory inputs, including visual greenery, fresher air, and reduced noise. These sensory cues reduce stress responses by freeing up attentional resources to focus on goal-directed activity [1, 4]. At the same time, interactive and complex-enhanced digital platforms, such as virtual reality (VR) and exergaming, for instance, have been introduced to stimulate interest, challenge the cognitive function, and raise the enjoyment through dynamic stimuli and task variability, especially among seniors and pre-frail adults [5–7].

To this end, the environment is not a neutral background; rather, it is a possible active agent that may modulate brain-related outcomes through autonomic and endocrine stress pathways, attentional and physiological control processes, and network-level neuroplasticity [2, 8, 9]. However, as mentioned, the environment is often not highlighted as the primary exposure in primary research to such an extent that exposure characteristics are not commonly reported in a standardized manner [10, 11]. Some trials exclusively measure either affective or restoration responses, while others measure physiological stress factors such as heart-rate variability and secretion of cortisol to better describe autonomic recovery after outdoor or supplemented physical activity sessions [12, 13]. A smaller subset has utilized functional near-infrared spectroscopy to demonstrate that exercise conducted in rich sensory contexts can change brain oxygenation patterns related to attentional control [14].

Expanding on this work, randomized and crossover trial designs of hiking versus treadmill walking, natural versus built environments, or other outdoor–indoor comparisons have revealed that the exercise environment may contribute to emotion, sense of restoration, executive function, dual-task gait, and enjoyment (although physiological measures have been found to vary more unevenly among studies [15–19]). Later randomized analyses extended this environmental approach to technology-assisted and immersive exercise in older adults, and the effects from combining interactive or dual-task modalities, which improve executive function, dual-task gait, and daily-function surrogates, are extended, particularly in community-living or cognitively fragile populations [20–25]. But inconsistencies in outcome and definitions of exposure limit our ability to examine at the population level whether the environmental context of exercise can systematically alter the impact of exercise on the brain.

### Research question

We investigate here whether the exercise environment itself offers additive benefits for brain-related outcomes in the presence of broadly similar physical activity parameters. In randomized or quasi-randomized contrasts of nature-based, outdoor, or immersive and interactive exercise and standard indoor or urban exercise, are participants able to show superior responses in cognitive and executive functions, affective and perceived restoration outcomes, stress-related autonomic or endocrine markers, or indices of brain activation and connectivity? By framing environmental context as the primary exposure, we evaluate whether it should be treated as a modifiable component of exercise prescription rather than as a secondary methodological factor.

### Objectives

Our objectives were threefold. First, a narrative synthesis of randomized evidence on the effects of natural environments and immersive contexts on brain-relevant outcomes across four domains: executive functions and global cognition, mood and perceived restoration, autonomic and endocrine physiology, and functional brain activation and connectivity. Second, to map results by design and comparator, and to summarize domain-specific findings at the subgroup level where possible (e.g., crossover versus parallel designs; heart-rate variability versus cortisol versus blood pressure), acknowledging that a full dose–response analysis was not practical. Third, to assess risk of bias based on current criteria, report study limitations, and recommend new methods for further trials, such as standardized reporting of environmental characteristics and immersion, and the use of core outcome batteries to facilitate cross-domain synthesis.

## Methods

### Eligibility criteria

We included randomized controlled trials that examined the effects of nature-based or technology-assisted physical activity on cognitive or mobility outcomes. The primary population of interest was older adults, operationally defined as individuals aged 60 years or older, or samples reporting a mean age of at least 60 years, including those with mild cognitive impairment.

Population descriptors such as frailty, cognitive vulnerability, or mild cognitive impairment were assigned according to the diagnostic or screening criteria reported in each original trial. No single frailty or cognitive framework was imposed across studies, given the heterogeneity of definitions used in the literature.

During screening, several trials involving younger adults were also identified. These trials were retained only for methodological completeness but were excluded from the primary synthesis, tables, and conclusions. They are summarized separately in Supplement S6. No restrictions were placed on sex or health status. Only peer-reviewed articles published in English were considered.

Eligible comparator conditions included sedentary controls, active controls (e.g., conventional exercise), or alternative digital interventions. Studies were required to report at least one cognitive or mobility outcome measured using validated tools.

### Search strategy

We conducted systematic searches in four electronic databases (PubMed, Web of Science, Scopus, and PsycINFO/EBSCO) from January 2010 to August 2025. Search strings incorporated controlled vocabulary and free-text terms for environments and immersive modalities, randomized designs, and brain-health outcomes. Core terms included “green exercise”, “outdoor exercise”, “forest”, “blue space”, “virtual reality”, “exergame”, “immersive”, “dual-task gait”, “executive function”, “brain activation”, and “prefrontal”, coupled with “randomized controlled trial” or “randomized crossover”. No additional study design filters were applied beyond randomization to avoid missing eligible records.

To ensure coverage, we also screened reference lists of all eligible trials and recent domain reviews and meta-analyses, and we forward-tracked key trials using citation alerts. When titles and abstracts suggested a potentially eligible comparison, but outcomes were not clearly described, we retrieved the full text. The full database-specific search strings, date limits, and record counts for each source are provided in Supplement S1.

### Study selection

Records identified from the databases were imported into a reference manager and then screened in two steps. After removal of duplicates, two reviewers independently screened titles and abstracts of the remaining records according to the predefined inclusion and exclusion criteria described in Eligibility criteria section. Full texts of potentially eligible articles were then retrieved and assessed in duplicate. Disagreements at either stage were resolved through discussion, with a senior reviewer available to adjudicate when needed.

A total of 1,532 records were retrieved, and 624 duplicates were excluded. Among the 908 unique records, all underwent title and abstract screening, and 128 full texts were assessed for eligibility. Twelve studies met all inclusion criteria and were included in the final synthesis. Trials involving younger adults that fulfilled other criteria were flagged during full-text screening and subsequently moved to Supplement S6, where they are documented for transparency but not incorporated into the primary synthesis.

**Fig. 1:**
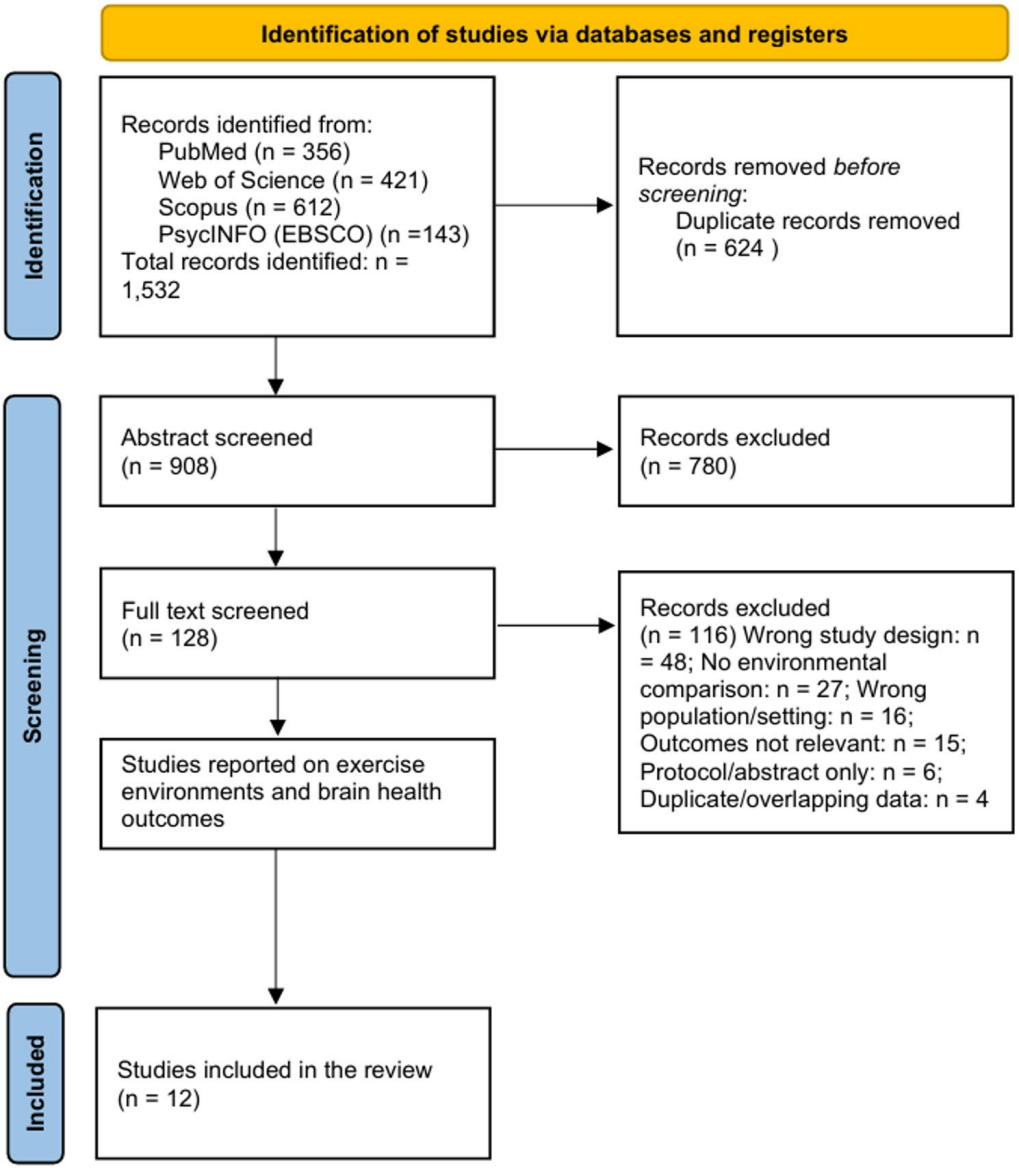
PRISMA flow diagram of study selection

Figure 1 presents the detailed selection process. Supplement S2 provides a structured list of excluded full-text records with one primary reason per record.

### Data extraction

We developed and piloted a standardized extraction form in two studies to maintain consistency. Extracted items included bibliographic details; country and setting; number of participants; and participant characteristics such as age (mean and, where available, standard deviation or range), sex distribution, and cognitive or health status. We also recorded the exercise prescription (mode, intensity target, duration, and frequency).

Because environment and immersion were considered the exposures of interest, when reported, we additionally extracted environmental and immersion characteristics: outdoor route or park types; indoor facility characteristics; weather; ambient noise and air quality; and, where applicable, virtual reality or exergaming specifications such as device model, field of view and refresh rate, motion-to-photon latency, interaction type, and presence or cybersickness scales. Data on comparator conditions, study design (parallel versus crossover; acute versus multi-session), outcome domains, and measurement instruments (for example affect scales, executive function tasks, dual-task paradigms, heart rate and heart-rate variability, cortisol, neuroimaging or functional near-infrared spectroscopy parameters), timing of assessments, adherence to protocols, co-interventions, adverse events, and main statistical results (group means and standard deviations, change scores, effect estimates, and p values) were also recorded.

The full extraction codebook and trial-level data fields for older-adult and mild cognitive impairment trials are available in Supplement S3. In data visualization, we only performed graphical displays and used digital plots where numerical data were required. If outcome statistics were not fully reported, we noted the limitations and summarized the direction of effect. Younger-adult randomized trials underwent the same extraction process but are presented only in Supplement S6 and do not contribute to the main synthesis. To further address variability in exposure reporting, environmental and immersion characteristics were additionally evaluated using a structured exposure fidelity framework, and each trial was rated according to the completeness and reproducibility of reported exposure features (Supplement S7). A standardized exposure reporting checklist was also developed to inform future trials (Supplement S8).

In this review, technology-assisted exercise is used as an umbrella term encompassing both virtual reality–based interventions and exergaming formats. Although these modalities share the use of digital interfaces to augment physical activity, they differ substantially in their degree of perceptual immersion, sensorimotor integration, and cognitive–motor task demands. Virtual reality–based interventions typically provide higher levels of immersive visual and sensory input and impose greater demands on multisensory integration and spatial processing, whereas exergaming includes a broader range of interactive exercise formats with variable immersion levels and task complexity. Accordingly, exposure characterization and subsequent synthesis considered immersion characteristics and task demands rather than assuming equivalence across technology-assisted modalities.

### Risk of bias assessment

Two reviewers evaluated the risk of bias independently for randomized trials at the study level using the RoB 2 tool. We examined five standard domains: bias arising from the randomization process, bias due to deviations from intended interventions, bias due to missing outcome data, bias in measurement of the outcome, and bias in selection of the reported result.

In contrasts between environmental or modality settings, we also considered the feasibility of blinding participants and outcome assessors, unequal exposure fidelity, route familiarity, social density, weather conditions, novelty effects, and, in crossover designs, period and carryover effects, and the adequacy of washout or counterbalancing. Judgments (low risk, some concerns, or high risk) were reached through consensus and summarized at the domain level in Table 1. Signaling-question responses with page-anchored justifications and the full RoB 2 tables are presented in Supplement S4.

**Table 1:** Domain-level risk-of-bias judgments for included randomized trials (RoB 2, abbreviated)

Study	Design	Randomization	Deviations	Missing data	Outcome measurement	Reported result	Overall
Niedermeier 2017 [15]	Crossover	SC	L	L	L	SC	SC
Laezza 2025 [16]	Parallel	SC	L	L	L	SC	SC
Ochiai 2025 [17]	Parallel	L	L	L	L	SC	L–SC
Zukowski 2022 [18]	Parallel (acute)	SC	L	L	L	SC	SC
Ahnesjö 2022 [19]	Crossover	L	L	L	L	SC	L–SC
Liao 2021 [20]	Parallel	L	L	L	L	SC	L–SC
Anderson-Hanley 2012 [21]	Cluster randomized	SC	L	L	SC	SC	SC
Liao 2019 [22]	Parallel	L	L	L	L	SC	L–SC
Eggenberger 2016 [23]	Parallel	SC	L	L	L	SC	SC
Liu 2022 [24]	3-arm parallel	L	L	L	L	SC	L–SC
Zhao 2022 [25]	3-arm parallel	SC	L	L	SC	SC	SC
Niedermeier 2017 [26]	Parallel	SC	L	L	L	SC	SC

Judgments follow the Cochrane RoB 2 tool (randomization, deviations from intended interventions, missing outcome data, outcome measurement, and selection of reported results). No trial was rated as high risk. Full signaling-question responses with page-anchored quotations appear in Supplement S4

*Abbreviations*: *L*  low risk of bias, *SC*  Some concerns, L–SC  Overall judgment spanning low risk to some concerns across domains

### Data synthesis

Because populations, intervention characteristics, and outcome measures were heterogeneous, we followed Cochrane guidance for reviews with substantial clinical and methodological variability and did not conduct a pooled meta-analysis. Although a subset of trials evaluated technology-assisted interventions and reported conceptually related outcomes, such as executive function and dual-task gait, these studies differed markedly in intervention modality (e.g., virtual reality treadmill training, exergaming, balance-focused exergames, and exergame-based Tai Chi), immersion level, cognitive-motor task demands, feedback structure, and training dose (acute versus multi-week interventions). These differences indicate that the interventions represent heterogeneous exposure packages rather than a single, well-defined treatment class suitable for quantitative pooling.

In addition, outcome operationalization was not directly comparable across trials. Executive function was assessed using diverse instruments, including Trail Making Test Part B, Stroop tasks, Digit Span, the Montreal Cognitive Assessment, and composite cognitive scores, while dual-task gait outcomes varied in both metrics (e.g., speed, variability, dual-task cost) and calculation methods. This variability limited the interpretability of standardized effect sizes and increased the risk that pooled estimates would be unstable or potentially misleading.

Accordingly, we adopted a structured narrative synthesis. This approach emphasizes the direction and consistency of effects, patterns across outcome domains, and population-specific responses rather than pooled point estimates. In crossover trials, we focused on within-subject comparisons across exposure conditions, with specific consideration of washout periods and counterbalancing sequences when reported. For parallel-group trials, we primarily examined between-group differences in change from baseline, where available, or differences at follow-up when change scores were not reported. For dual-task paradigms, gait and cognitive outcomes were extracted simultaneously and interpreted as integrated indicators of cognitive–motor loading.

In randomized crossover trials, data extraction and interpretation followed a prespecified, conservative approach. We did not restrict analyses to first-period data. Instead, where available, we prioritized within-subject (paired) comparisons that directly contrasted exposure conditions within the same participants, consistent with the methodological rationale of crossover designs. When paired differences or condition-specific contrasts were explicitly reported by the original authors, these data were extracted and interpreted descriptively. Particular attention was paid to washout periods and counterbalancing sequences. Trials reporting adequate washout and no evidence of carryover effects were interpreted using within-subject contrasts. In contrast, when potential carryover effects were noted or when washout adequacy was unclear, findings were summarized qualitatively with emphasis on the direction and consistency of effects rather than on magnitude.

Planned descriptive subgrouping separated acute versus training interventions and mental status categories (healthy, frail, or mild cognitive impairment), which facilitated interpretation and potential translation. Environmental and immersion descriptors extracted in Data extraction section were used to contextualize exposure fidelity and to aid understanding of heterogeneity in findings, with structured summaries reported in Supplement S5.

Certainty-of-evidence judgments and summary-of-findings tables for the primary outcome families in older adults and individuals with mild cognitive impairment were generated using the GRADE approach. Randomized trials involving younger adults were retained for methodological completeness but are reported only in Supplement S6 and were not incorporated into the primary synthesis or conclusions.

### Additional methodological considerations

Because environment and immersion are complex, multicomponent exposures, we treated each trial as an exposure package rather than attempting to isolate single environmental elements. In interpreting findings, we therefore considered not only nominal contrasts (for example, outdoor versus indoor, virtual reality versus conventional training), but also the plausibility of exposure fidelity, potential novelty effects, and the feasibility of replicating the exposure in clinical or community programs.

For crossover designs, we took account of possible carryover and residual adaptation even when washout or counterbalancing was described, and we interpreted small effects cautiously if exposure sequences were not clearly reported. For dual-task outcomes, we focused on patterns that were coherent across cognitive and gait components, recognizing that changes confined to a single component alone may be less meaningful for fall risk or functional translation.

These considerations guided the narrative synthesis and the emphasis placed on specific contrasts, without altering the underlying extraction or risk-of-bias assessments described above. This review followed PRISMA 2020 reporting standards. A completed PRISMA 2020 checklist is provided as Additional File 1.

## Results

### Study characteristics

The twelve randomized trials included in this review involved participants ranging from healthy young adults to frail older adults and individuals with mild cognitive impairment. For the purposes of the primary synthesis, eight trials in older adults and those with mild cognitive impairment formed the core evidence base, with mean ages in these samples ranging approximately from 65 to 82 years. Four trials in healthy young or middle-aged adults are described separately for completeness and do not inform the main conclusions.

Five studies compared outdoor and nature-based activity with indoor or built-environment exercise formats [15–17, 19, 26], and seven compared immersive or interactive methods (exergaming or virtual reality) with standard exercise [18, 20–25]. Intervention length varied from a single acute bout to twelve-week training programs. Across these trials, primary outcome families included affective valence and arousal, perceived restorativeness, executive and working-memory functions, dual-task gait measures, prefrontal activation assessed by functional near-infrared spectroscopy (fNIRS), and physiological markers such as heart rate (HR), heart-rate variability (HRV), salivary cortisol, and mucosal immunity.

To keep the main synthesis older-adult focused, Supplement S6 presents young- and middle-aged adult trials that met the design criteria but did not include older or mild cognitive impairment populations. The numbering of references in all tables follows the master reference list [15–26]. Tables 2 and 3 summarize designs, exposure contrasts, populations, and cognition- and mobility-related outcomes for older adults and those with mild cognitive impairment, enabling rapid comparison across trials, while physiological and affective endpoints are described in the text with cross-references to Supplements S4–S6 where required. In trials employing dual-task gait paradigms, the specific cognitive tasks paired with walking varied across studies and included working memory, attention, and executive function challenges. A detailed description of the cognitive tasks used in each study is provided in Supplement S10.

**Table 2:** Cognitive outcomes in older adults and MCI: randomized comparisons of nature-based or technology-assisted exercise versus dose-matched indoor training

[Ref]	Study (Year)	Population/ age (mean ± SD)	Design & Dose	Environment / Modality Contrast	Primary Outcome Domains	Main Findings (Direction)	Notes
[18]	Zukowski et al., 2022	Older adults (71.6 ± NR)	Parallel Randomized controlled trial (RCT); acute (30 min)	VR-augmented treadmill vs. conventional treadmill	Gait, cognition (reaction/accuracy), dual-task	Both improved; VR ≈ conventional acutely	Suggests the need for repeated dosing
[20]	Liao et al., 2021	Frail older adults (81.7 ± NR)	Parallel RCT; training (≈ 12 weeks)	Exergaming vs. combined exercise	Global & executive cognition; fNIRS (prefrontal cortex (PFC))	**Exergaming ↑ MoCA** (**montreal cognitive assessment) /memory; PFC activation ↓ (efficiency ↑)**	Neural efficiency signal
[21]	Anderson-Hanley et al., 2012	Community-dwelling older adults (78.65 ± NR)	Cluster RCT; training	Exergaming/cybercycle vs. conventional	Executive function, cognition	**Exergaming ↑ executive outcomes**	Early seminal exergaming trial
[22]	Liao et al., 2019	Older adults with MCI (74.3 ± NR)	Parallel RCT; training	VR-based physical-cognitive vs. control	Executive function; dual-task gait	**VR ↑ exec function & dual-task gait**	Cognitively vulnerable subgroup
[23]	Eggenberger et al., 2016	Older adults (74.9 ± NR)	Parallel RCT; training	Exergame + balance vs. balance control	Executive function; fNIRS during walking	**Exec function ↑; PFC modulation**	Task-congruent neural change
[24]	Liu et al., 2022	Older adults with MCI (73.73 ± NR)	Parallel RCT; training	Exergaming-based Tai Chi vs. traditional Tai Chi vs. control	Global cognition; dual-task gait	**Exergaming-based Tai Chi (EXER-TC) ↑ global cognition vs. TC/ctrl**; dual-task gait ∼	Population with MCI
[25]	Zhao et al., 2022	Older adults (65.4 ± NR)	Parallel RCT; training	Exergame training vs. conventional	Working memory, executive function	**Exergame ↑Working Memory / Executive Function (WM/EF)**	Sustainability-journal RCT

Detailed notes for Tables 2 and 3 appear below Table 3. Population categories reflect study-specific diagnostic or screening criteria as reported by the original authors; detailed operational definitions are provided in Supplement S9

Tables 2 and 3 follow an older-adult priority rule. In mixed-age randomized trials, summary rows present outcomes for participants aged ≥60 years or those with MCI, while younger-adult data are collated in Supplement S6 for completeness. Arrows indicate the direction of effect (↑ improvement for the intervention relative to the comparator; → no clear between-group difference at the primary time point). Bolded entries highlight changes interpreted as clinically meaningful or central to the trial’s main conclusions, for example, improvements in global or executive cognition, dual-task gait, or other outcomes emphasized by the original authors. Executive-function assessments commonly include the Trail Making Test Part B (TMT-B), Stroop tasks, Digit Span backward, verbal fluency, and the Digit Symbol Substitution Test (DSST); composite definitions and component tests are described in Supplement S3

Exposure fidelity coding records, for nature-based arms, route and terrain characteristics, and greenness or blue-space descriptors, and for technology-assisted arms, device type, immersion level, and interactivity, with the coding scheme detailed in Supplement S1/S6. Domain-level risk of bias is summarized using the RoB 2 tool, with item-level justifications and page-anchored quotations provided in Supplement S4. Outcomes are reported in their native units, with higher scores indicating better performance unless otherwise specified; where necessary, scales were reoriented to harmonize interpretation. Extraction fields for all trials included in these tables are available as a machine-readable file in Supplement S3 (CSV)

**Table 3:** Mobility and dual-task gait outcomes in older adults and MCI: randomized comparisons of nature-based or technology-assisted exercise versus dose-matched indoor training

[Ref]	Study (Year)	Population/ age (mean ± SD)	Design & Dose	Environment / Modality Contrast	Primary Outcome Domains	Main Findings (Direction)	Notes
[18]	Zukowski et al., 2022	Older adults (71.6 ± NR)	Parallel RCT; acute (30 min)	VR-augmented treadmill vs. conventional treadmill	Gait, cognition (reaction/accuracy), dual-task	Both improved; VR ≈ conventional acutely	Suggests the need for repeated dosing
[22]	Liao et al., 2019	Older adults with MCI (74.3 ± NR)	Parallel RCT; training	VR-based physical-cognitive vs. control	Executive function; dual-task gait	**VR ↑ exec function & dual-task gait**	Cognitively vulnerable subgroup
[24]	Liu et al., 2022	Older adults with MCI (73.73 ± NR)	Parallel RCT; training	Exergaming-based Tai Chi vs. traditional Tai Chi vs. control	Global cognition; dual-task gait	**EXER-TC ↑ global cognition vs. TC/ctrl**; dual-task gait ∼	Population with MCI

Tables 2 and 3 follow an older-adult priority rule. In mixed-age randomized trials, summary rows present outcomes for participants aged ≥60 years or those with MCI, while younger-adult data are collated in Supplement S6 for completeness. Arrows indicate the direction of effect (↑ improvement for the intervention relative to the comparator; → no clear between-group difference at the primary time point). Bolded entries highlight changes interpreted as clinically meaningful or central to the trial’s main conclusions, for example, improvements in global or executive cognition, dual-task gait, or other outcomes emphasized by the original authors. Executive-function assessments commonly include the Trail Making Test Part B (TMT-B), Stroop tasks, Digit Span backward, verbal fluency, and the Digit Symbol Substitution Test (DSST); composite definitions and component tests are described in Supplement S3

Exposure fidelity coding records, for nature-based arms, route and terrain characteristics, and greenness or blue-space descriptors, and for technology-assisted arms, device type, immersion level, and interactivity, with the coding scheme detailed in Supplement S1/S6. Domain-level risk of bias is summarized using the RoB 2 tool, with item-level justifications and page-anchored quotations provided in Supplement S4. Outcomes are reported in their native units, with higher scores indicating better performance unless otherwise specified; where necessary, scales were reoriented to harmonize interpretation. Extraction fields for all trials included in these tables are available as a machine-readable file in Supplement S3 (CSV)

### Acute versus training effects

At the outset, it is important to clarify that intervention duration (acute versus multi-week training) and exposure type (nature-based versus technology-assisted exercise) represent distinct but intersecting analytical dimensions. In the current evidence base, nature-based interventions are more commonly examined as single-session or short-term exposures, whereas technology-assisted interventions are more frequently implemented as repeated, multi-week training programs. Consequently, patterns observed across acute versus training comparisons partly reflect this distribution of study designs rather than a pure effect of exposure type alone. Accordingly, the contrasts presented in this section are organized primarily by temporal scale (acute versus training effects), while interpretation explicitly considers the characteristics of the exercise context. Outcome patterns should therefore be understood as arising from the combined influence of exposure features and intervention duration, rather than being attributed solely to one dimension.

There were apparent differences between acute and training effects across the included randomized trials. In acute exposure studies, nature-based activities such as green walking, cycling, or hiking generally produced greater improvements in affect, perceived restoration, and exercise enjoyment than indoor or urban comparators [27–31]. These findings indicate robust short-term psychological and motivational benefits of natural settings rather than direct, immediate physiological or cognitive changes. The only single-session virtual reality exposure showed no clear advantage over traditional treadmill walking in perceived exertion or cognitive outcomes [32, 33].

In contrast, training-based interventions that delivered multiple sessions of immersive or technology-assisted exercise (for example, virtual reality balance training, exergaming, or interactive multimodal programs) elicited more pronounced adaptations [34, 35], particularly in executive function, attention, and dual-task gait among older adults and individuals with mild cognitive impairment [36–39]. These training-related gains typically exceeded those seen after single sessions and highlight the importance of repeated exposure, progressive task difficulty, and multisensory engagement.

Acute and training results are therefore complementary rather than mutually exclusive. Acute green exercise reliably enhances psychological states and motivation, while multi-week immersive or interactive training appears necessary to improve cognitive–motor performance, especially among populations vulnerable to cognitive decline. Outcome- and population-specific results are further detailed in Older adults and cognitively vulnerable groups section and Cognition and executive function / dual-task gait section.

#### Acute interventions

Across acute trials, nature-based or outdoor programs consistently yielded more favorable affective responses, perceived restoration, and exercise enjoyment than dose-matched indoor or built-environment conditions [15–17, 19, 26]. These benefits were observed even when ratings of exertion were equivalent on average, suggesting that the environmental context, rather than lower physical load, is the primary driver of the psychological response. By contrast, autonomic and endocrine markers showed smaller and less consistent added value of natural conditions. Heart rate and HRV profiles differentiated settings only modestly, and salivary cortisol responses appeared to be driven more strongly by the exercise bout itself and the sampling schedule than by environment alone [16, 19, 26].

The sole acute virtual reality contrast did not provide compelling evidence that a single VR-based session outperformed conventional treadmill exercise in terms of perceived exertion or cognitive performance, which supports the notion that one immersive exposure is unlikely to confer additional short-term benefits beyond standard training in older adults [18–25]. Overall, acute findings indicate rapid and consistent improvements in affect and restoration following nature exposure, but more ambiguous evidence for immediate physiological or cognitive change after a single session. Population- and outcome-specific details are presented in Population subgroups section and Outcome families section.

#### Training interventions

For multi-week interventions, immersive and interactive training formats, including exergaming and virtual reality–enhanced balance or gait programs, produced more robust adaptations in older and cognitively vulnerable adults [18, 20–25]. Compared with conventional exercise of similar frequency and duration, these programs frequently yielded greater improvements in executive function, attention, and dual-task gait, and some studies reported enhanced balance and functional mobility. Effects were especially evident in frail older adults and participants with mild cognitive impairment, who began with lower baseline performance and higher dual-task costs [18, 20–25, 36–39].

Nature-based or outdoor training programs in older adults were fewer in number but directionally positive. Over several weeks, park-based or outdoor walking interventions with specific goals improved mood and perceived restoration and, in some cases, walking speed or balance when task-specific challenges were incorporated [15–17, 19, 26]. Taken together, the pattern of acute and training findings suggests that nature-based exposure is a potent, low-cost lever to improve affective experience and motivation, while immersive and interactive formats require repeated sessions to generate cognitive and dual-task gait and mobility gains. The distribution of these effects across subgroups and outcome families is detailed in Population subgroups section and Outcome families section.

### Population subgroups

#### Healthy adults

In randomized controlled trials conducted in healthy young to middle-aged adults, nature exposure produced consistent affective and restorative benefits, with a more complex pattern for endocrine and autonomic responses. In a randomized crossover trial of 42 subjects, an approximately 3-hour alpine hiking session resulted in larger increases in affective valence and arousal and decreases in fatigue and anxiety than treadmill walking of matched duration [15]. In a similar crossover experiment with comparable design and duration, salivary cortisol declined after both outdoor hiking and indoor treadmill walking relative to a non-exercise control, with no marked differences between settings in cortisol or blood pressure; HRV measured 5 min after exercise suggested that the exercise stimulus, rather than the setting, predominated in shaping the acute endocrine response [26].

A more recent parallel RCT comparing green, urban, and indoor exercise settings with fixed exercise doses demonstrated that green environments consistently produced higher perceived restorativeness, more positive emotions, lower negative affect, stronger intention to return to exercise, and slightly lower perceived exertion, whereas physiological readouts (cortisol, HR, HRV) indicated more favorable recovery in green environments compared with urban or indoor settings but with variable significance across indices [16]. Extending this work, a 3 × 3 randomized crossover study in adults exercising at a fixed Rating of Perceived Exertion (RPE 11–13) on cycle ergometers in indoor, simulated-outdoor, and outdoor conditions revealed no statistically significant environmental effects on power output or HR (both *p* ≈ 0.07). However, trends in outdoor conditions were consistent with minor intensity-related differences where workload control was imperfect [19].

Together, these trials indicate that the affective and psychological benefits of natural settings are robust and medium to large in healthy adults, while physiological stress markers such as cortisol, HRV, and blood pressure show mixed or small setting effects at a matched exercise dose [15, 16, 19, 26]. Design features such as assessment timing for cortisol, workload matching, and inclusion of downhill components likely moderate the detectability of setting effects on physiology, whereas emotional effects appear more consistent across concurrent and crossover designs [15, 16, 19, 26]. These findings are presented for completeness and context but do not contribute directly to the core geriatric conclusions.

#### Older adults and cognitively vulnerable groups

Trials in community-dwelling and frail older adults and those with mild cognitive impairment demonstrated the most evident functional benefits. Across several weeks, multicomponent and balance-focused exergaming or virtual reality programs were associated with significant gains in executive tasks, attention, and dual-task gait that often exceeded those observed with conventional balance or strength training [18, 20–25]. Immersive or semi-immersive balance training in frail older adults improved step accuracy, gait adaptability, and performance under cognitively demanding conditions, supporting a potential role in reducing fall risk [20–22].

Task switching, processing speed, and stability in dual-task walking were consistently enhanced by technology-assisted training in participants with mild cognitive impairment [23–25]. None of these trials indicated deterioration in cognition or mobility relative to conventional exercise. Evidence for nature-based interventions in these at-risk demographics is more limited. However, outdoor or park-based walking with specific aims appears to improve mood and perceived restoration and may confer modest mobility benefits without apparent harm [15–17, 19, 26]. In general, the most pronounced cognitive–motor benefits for older and cognitively vulnerable populations appear to stem from structured, multisensory, progressively challenging interventions. At the same time, nature-based formats provide motivational and affective support that may be leveraged alongside task-specific training.

### Outcome families

#### Affective, restorative, and mobility outcomes in nature-based versus indoor conditions

In the five randomized trials comparing nature-based or outdoor exercise with indoor or built-environment formats [15–17, 19, 26], the most consistent advantages were observed for affective valence, enjoyment, and perceived restoration. Participants reported higher post-session pleasure, less arousal-related discomfort, and stronger perceptions of mental well-being or restoration after outdoor experiences, even when exercise intensity and duration were matched. These affective and restorative responses were seen in both healthy adults and older participants, underscoring the contribution of environmental context to subjective experience beyond the primary physiological effects of exercise.

Findings on gait and mobility were more limited. A subset of trials evaluated simple gait outcomes or short walking tasks, and environmental differences produced only modest and inconsistent benefits. Available data suggest that outdoor walking may subtly influence pace or step regularity through different sensory cues, but these differences did not consistently reach statistically or clinically meaningful thresholds in older-adult samples.

Physiological effects were heterogeneous and generally small. Heart rate and heart-rate variability tended to reflect the exercise dose more strongly than the environment, and salivary cortisol trajectories typically followed expected exercise-related patterns with minimal additional modulation by setting [16, 17, 19, 26]. Preliminary evidence from a forest-exposure trial suggested short-term increases in salivary immunoglobulin A (IgA), but this finding requires replication [17]. Overall, nature-based exercise consistently improves affect, enjoyment, and perceived restoration, with more variable and modest effects on mobility and physiological markers. A consolidated summary of nature-based versus indoor exercise across affective, restorative, physiological, and mobility endpoints is presented in Table 4.

**Table 4:** Effects of nature-based versus indoor exercise on affective and physiological outcomes in older adults

Domain / outcome	Representative studies	Direction of effect (outdoor vs. indoor)	Approximate effect magnitude (descriptive)	Certainty (qualitative)
Affective valence and enjoyment	[15, 16, 19]	↑ Favors outdoor	Moderate to large improvement	Moderate to high
Perceived restoration and intention to repeat	[16, 19]	↑ Favors outdoor	Moderate improvement	Moderate
Autonomic indices (HR and HRV)	[16, 19]	↑ / ↔ Mixed	Small and inconsistent changes	Low (mixed effects)
Cortisol / stress markers	[26]	↔ No clear difference	No consistent effect	Low
Immune function (salivary IgA)	[17]	↑ Favors outdoor	Small to moderate improvement	Moderate

Approximate effect magnitudes are descriptive summaries derived from individual trials to facilitate comparison across outcome domains. These values do not represent pooled estimates and should not be interpreted as results of a formal meta-analysis

*Abbreviations*: *SD*  Standard deviation, *CI*  Confidence interval, *HR*  Heart rate, *HRV*  Heart-rate variability, *rMSSD*  Root mean square of successive differences (a parasympathetic HRV index), *IgA*  Immunoglobulin A (indicator of mucosal immune activity)

Direction of effect symbols: ↑ = favors outdoor or nature-based exercise; ↔ = no clear difference between outdoor and indoor conditions; Mixed = heterogeneous findings across trials

#### Cognition and executive function / dual-task gait

Across randomized trials, cognitive outcomes showed the strongest improvements in executive domains and dual-task gait and mobility. Multi-week exergaming and virtual reality programs in older adults and those with mild cognitive impairment produced moderate-to-large gains in executive performance, including task switching, inhibition, and working memory, compared with conventional exercise [18, 20–25]. These cognitive improvements were often accompanied by faster gait speed, reduced stride-time variability, and better stability during concurrent cognitive tasks in dual-task paradigms [20–25]. Effects were frequently greater in frail or cognitively vulnerable groups that started with lower baseline performance and higher dual-task costs.

By contrast, acute nature-based trials mainly demonstrated benefits in affective and restorative outcomes, with limited evidence of immediate cognitive improvement in older adults [15–17, 19, 26]. Where cognition was assessed following a single outdoor or nature exposure, effect sizes tended to be small and heterogeneous, suggesting that acute exposure to nature alone may not be sufficient to alter executive performance in this age group. Similarly, the single-session virtual reality contrast did not yield clear cognitive benefits over typical treadmill training, reinforcing the view that cognitive–motor adaptations arise through repeated practice and escalating challenge rather than single-dose exposure [18–25].

Taken together, these findings indicate that for older and cognitively vulnerable individuals, executive functions and dual-task gait respond most strongly to structured, interactive, repeated training that integrates explicit cognitive demands into movement tasks. Nature-based formats are valuable for mood and motivation but may need to be combined with task-specific challenges or multi-session dosing to achieve comparable cognitive–motor advantages. Table 5 summarizes findings comparing immersive and interactive (virtual reality or exergaming) versus conventional exercise on executive function, global cognition, dual-task gait, prefrontal activation, and mood outcomes.

**Table 5:** Effects of technology-assisted (immersive / interactive) versus conventional exercise on cognitive and mobility outcomes in older adults

Domain / Outcome	Representative studies	Direction of effect (immersive/interactive vs. conventional)	Approximate effect magnitude (descriptive)	Certainty (qualitative)
Executive function and working memory	[20–25]	↑ Favors immersive/interactive	Small to moderate improvement	Moderate to high
Global cognition (MoCA or MMSE)	[20, 24]	↑ Favors immersive/interactive	Clinically meaningful improvement	Moderate
Dual-task gait (speed and variability)	[18, 22–25]	↑ Favors immersive/interactive	Moderate improvement in dual-task performance	Moderate
Prefrontal activation during walking (fNIRS)	[20, 23]	↓ (activation ↓ = efficiency ↑)	Small to moderate reduction in activation (greater neural efficiency)	Moderate
Mood and enjoyment (primarily acute post-session)	[18, 20]	↑ Favors immersive/interactive	Small and inconsistent improvement	Low to moderate

Approximate effect magnitudes are descriptive summaries derived from individual trials to facilitate comparison across outcome domains. These values do not represent pooled estimates and should not be interpreted as results of a formal meta-analysis. ↓ prefrontal activation is interpreted as greater neural efficiency at comparable task performance. Certainty ratings (low, moderate, high, or ranges such as low to moderate) reflect qualitative GRADE-informed judgements that integrate risk of bias, inconsistency, imprecision, and indirectness, as detailed in Supplement S5. Mood and enjoyment reflect mainly acute post-session ratings in the contributing trials

*Abbreviations*: *SD*  Standard deviation, *CI*  Confidence interval, *MoCA*  Montreal Cognitive Assessment, *MMSE*  Mini-Mental State Examination, *fNIRS*  Functional near-infrared spectroscopy, *VR*  Virtual reality

#### Autonomics and endocrine physiology

Across the trials reporting autonomic and endocrine effects, environmental influences were generally smaller and less consistent than the affective benefits of nature-based sessions. Heart rate and HRV indices sometimes favored outdoor or forest conditions over indoor or urban comparators, with patterns suggestive of greater vagal influence after nature exposure, but effect sizes were typically small to moderate and not uniform across studies [16, 17, 19, 26]. In many comparisons, the primary signal appeared to be the exercise bout itself rather than the environment, and acute changes in HR or HRV often returned toward baseline within a short recovery period.

For endocrine markers, salivary cortisol responses were primarily determined by exercise intensity, sampling timing, and diurnal variation, with limited additional effect of nature-based or technology-assisted formats beyond these factors [16, 19, 26]. One forest-walking trial reported preliminary evidence that nature exposure may support mucosal immunity through favorable changes in secretory immunoglobulin A compared with indoor walking [17], but this finding has not yet been replicated. Overall, current autonomic and endocrine data suggest that while context may modulate stress physiology, evidence is less robust and more heterogeneous than for affective and cognitive–motor outcomes, and longer protocols with more comprehensive sampling will be needed to clarify these pathways.

#### Functional brain activation and efficiency

In neuroimaging-integrated fNIRS studies of immersive or interactive training, there was a consistent pattern of improved neural efficiency. Reduced prefrontal recruitment was accompanied by stable or enhanced task performance, suggesting more efficient cortical processing. In frail older adults, a 12-week randomized trial combining exergaming with physical activity showed significantly attenuated dorsolateral prefrontal cortex (DLPFC) activation during executive tasks alongside gains in global cognition and memory compared with an exercise-only group [20]. The decline in DLPFC hemodynamic response did not correspond to reduced accuracy or speed, supporting the interpretation that interactive training can reduce cortical demand for a given task while enhancing cognitive-motor performance.

In a randomized trial of balance exergame training in community-dwelling older adults, exercise enhanced executive function and altered the relationship between performance and fNIRS indices. Changes in prefrontal oxygenation relative to controls were observed during post-training gait and dual-task walking, consistent with task-congruent cortical adaptation in networks involved in attentional control and gait regulation [23]. Collectively, these findings suggest that simultaneous cognitive and motor training can be learned and consolidated in older adults, with improvements in visuomotor processing and coordination accompanied by more efficient prefrontal hemodynamic responses.

A notable gap in the current corpus is that no outdoor versus indoor trials have incorporated simultaneous neuroimaging, so it remains unclear whether nature exposure exerts comparable effects on online cortical recruitment during exercise or cognitive-motor tasks. Addressing this gap using standardized fNIRS protocols, including harmonized executive paradigms, consistent channel and region-of-interest reporting, and Δ[HbO₂]/Δ[HHb] outputs, would allow broader cross-domain generalizations to be made regarding the neural pathways linking affective and autonomic benefits of nature exposure [20, 23].

### Comparator and exposure integrity

For internal validity, comparator and exposure integrity are as important as exercise dose. In the included trials, investigators generally attempted to match frequency, duration, and target intensity across arms; however, practical differences in cadence, route complexity, visual stimulation, or task demands likely contributed to the observed physiological or cognitive responses. Indoor comparators often involved less varied scenery, shorter corridors, or more constrained balance tasks than outdoor or virtual reality conditions, even when nominal exercise prescriptions were identical, which may have generated additional variability, challenge, or engagement in experimental arms.

Exposure fidelity, meaning what participants actually experienced in each condition, was not consistently reported. Few nature-based trials quantified greenness, noise, air quality, or weather in a standardized manner, and most technology-assisted trials did not adequately characterize display features, latency, or interaction modes that influence immersion and attentional capture. From an exposure-science perspective, the exposome literature suggests that incomplete characterization of sensory and contextual environments can obscure dose–response relationships and limit reproducibility [11, 19]. Future randomized trials would be strengthened by treating environmental and immersive attributes as measurable exposures and by reporting comparator content clearly so that outcome differences can be linked to specific, reproducible aspects of the exercise context.

### Risk of bias

All trials were randomized, although details of allocation concealment were variably reported. Participant blinding was not feasible due to visible environmental or device-related differences, but several studies used blinded assessors for cognitive outcomes [20–22, 24]. Sample sizes were modest, typically ranging from about 25 to 60 participants, and while crossover designs helped reduce inter-subject variance, they sometimes lacked clear documentation of washout adequacy. Outcome reporting was generally complete, but descriptions of environmental and exposure fidelity were inconsistent, which limited comparability across trials.

Overall, the risk of bias ranged from low to moderate, with primary concerns related to performance bias and incomplete exposure characterization. Full RoB 2 domain-level signaling-question assessments with page-anchored justifications are provided in Supplement S4. These appraisals were considered alongside the summary of findings in Tables 4 and 5 and the certainty ratings in Supplement S5 when interpreting the consistency and robustness of the evidence.

## Discussion

### Principal findings

Outdoor and natural exercise consistently elicited moderate improvements in affective valence, arousal, enjoyment, and perceived restoration compared with matched indoor or built environments across randomized crossover and parallel trials [15–17, 19]. When assessed, intention to repeat exercise was also higher following nature exposure, indicating a potential motivational pathway supporting sustained participation [16]. In contrast, physiological stress markers exhibited more heterogeneous patterns. Although some heart rate and heart rate variability indices favored natural settings, these effects were generally modest and index-specific, while acute cortisol responses appeared more strongly related to the exercise bout itself than to environmental exposure [16, 19, 26]. A forest walking trial additionally suggested potential benefits for mucosal immunity, indicating that environmental context may influence host immune responses beyond subjective and autonomic outcomes [17].

For immersive and interactive exercise formats, including virtual reality–based interventions and exergaming, the majority of multi-week trials demonstrated improvements in executive function, working memory, and dual-task gait. Converging evidence from functional near-infrared spectroscopy indicated reduced prefrontal recruitment at comparable levels of task performance, consistent with enhanced neural efficiency [20–25]. These effects appeared dose sensitive. Single-session virtual reality exposures produced, at most, modest gains in gait or cognitive speed and did not consistently outperform conventional treadmill exercise, whereas programs delivered over approximately 12 weeks to frail or cognitively vulnerable older adults yielded broader and more robust adaptations [18, 20–25]. Collectively, these findings suggest that repeated exposure to cognitively demanding motor tasks represents a plausible active ingredient for executive and mobility adaptations in aging populations.

Although the cognitive benefits of conventional aerobic and resistance training are well established, the present review extends this literature by emphasizing the role of exercise context and delivery mode as modulators of cognitive and mobility outcomes in older adults. Rather than treating exercise as a unitary stimulus, this synthesis highlights how the experiential and task-related characteristics of exercise may introduce distinct mechanistic pathways. Technology-assisted exercise formats, including virtual reality–based interventions and exergaming, may confer theoretical advantages by increasing cognitive–motor integration demands, engaging executive control processes through interactive and adaptive task constraints, and providing immediate multimodal feedback that supports neural efficiency and learning. In contrast, nature-based exercise appears to preferentially influence affective and motivational pathways, including perceived restoration, enjoyment, and intention to continue exercise, which are critical determinants of long-term adherence in older populations. Together, these observations suggest that technology-assisted and nature-based modalities offer complementary benefits that extend beyond traditional training paradigms and may be strategically combined to optimize cognitive and functional health in aging.

Baseline cognitive function also emerged as an important moderator of intervention responsiveness. Across the included trials, participants with mild cognitive impairment, frailty, or elevated baseline dual-task costs tended to exhibit larger and more consistent gains in executive function and dual-task gait following multi-week, technology-assisted training. These individuals may derive particular benefit from interventions that impose structured cognitive–motor integration demands and provide external feedback to support executive control and learning. In contrast, cognitively healthy older adults more consistently demonstrated improvements in affective state, perceived restoration, and exercise enjoyment, especially in response to nature-based exercise. For this population, baseline cognitive capacity may be sufficient to perform physical tasks without additional cognitive scaffolding, rendering affective and motivational pathways more salient drivers of engagement and adherence. Taken together, these patterns indicate that baseline cognitive profile should inform intervention selection and personalization rather than assuming uniform effects across cognitive strata.

An additional consideration concerns the temporal scale of the underlying neurobiological mechanisms. Available evidence suggests that nature-based and technology-assisted exercise operate through distinct but complementary time-dependent pathways. Nature-based exercise appears to preferentially engage acute mechanisms, including reductions in psychological stress, modulation of affective state, and autonomic regulation, which may manifest rapidly following exposure and facilitate immediate improvements in mood, perceived restoration, and exercise enjoyment. These acute responses may lower psychological and physiological barriers to participation and support sustained engagement. In contrast, technology-assisted exercise interventions, particularly when delivered over multiple weeks, are more likely to elicit longer-term neurobiological adaptations. Repeated exposure to cognitively demanding, interactive, and feedback-rich tasks may promote neuroplastic processes, enhanced neural efficiency, and strengthened cognitive–motor integration, reflected in gains in executive function and dual-task mobility. Distinguishing between these acute and long-term mechanisms provides a more comprehensive framework for understanding how different exercise modalities contribute to cognitive and functional health in aging and highlights the potential value of integrating both approaches within multimodal intervention strategies. These interpretations reflect converging patterns observed across the included randomized trials rather than evidence from any single study [15–26].

From a clinical perspective, the functional relevance of observed effects should be interpreted in relation to established minimal clinically important difference thresholds commonly applied in geriatric research. For global cognition, improvements of approximately 1.22 to 3 points on the Montreal Cognitive Assessment are generally considered clinically meaningful, while changes in usual gait speed of 0.05 to 0.1 m/s and reductions in dual-task cost of 10 to 20% are often used as benchmarks for functional relevance. Across the included trials, multi-week, technology-assisted interventions frequently reported gains in executive function and dual-task gait that approached or overlapped with these clinically meaningful ranges, particularly among older adults with mild cognitive impairment or elevated baseline dual-task costs. In contrast, single-session or short-term nature-based interventions primarily improved affective state, motivation, and perceived restoration. Although these outcomes are not captured by conventional minimal clinically important difference metrics, they represent clinically relevant facilitators of exercise adherence and sustained participation in older adults.

Importantly, minimal clinically important difference thresholds were applied as interpretive reference points rather than strict criteria for clinical effectiveness. Heterogeneity in outcome measures, reporting of change scores, and intervention designs precluded formal responder analyses based on these benchmarks. Future trials should prospectively incorporate minimal clinically important difference criteria and standardized reporting practices to enable more definitive assessments of clinical significance.

Overall, the trial-level evidence indicates that environmental context and exercise modality are relevant determinants of cognitive and mobility outcomes in older adults. Nature-based exercise reliably enhances affective and restorative responses and intention to return, whereas immersive and interactive training most consistently improves executive control and dual-task gait. Nevertheless, protocol heterogeneity, variability in exposure fidelity, and outcome measurement diversity limit the strength of inference and support the low to moderate certainty ratings for executive function, dual-task gait, affective outcomes, and physiological or neurophysiological measures summarized in Supplement S5 [15–26].

### Mechanistic considerations

Natural contexts offer more diverse sensory inputs, such as fractally shaped green visual fields, relatively low-maintenance soundscapes, and cleaner air that are more amenable to attention-restoration schemes and lighten the load on directed attention and promote mental health [27–31, 40]. Throughout the trials and associated experiments, restoration perception and positive affect often closely correspond, both of them correlating with higher motivation to perform the same stimulus again [27–31, 39]. Efforts to emulate those benefits indoors or digitally demonstrate that stress modulation is still feasible, but effect amplitude and robustness have been found to correspond with the fidelity of incoming sensory stimuli and the context of the task, namely the least natural “nature” exposures (low parallax motion stimuli, simple sound, olfactory cues are absent), generating smaller or less reliable effects [13]. Such mechanisms can help to explain why, in our meta-analysis, nature-based conditions are found to have strong affect- and restorative-enhancing effects more robustly than our findings on physiological performance and impact markers, despite a more modest and variable contribution to physiological indicators.

Interactive and immersive exercise adds a new ingredient to that mix: active and focused cognitive load plus motion. Through the integration of goal-oriented choices, fast visuomotor updating, feedback, and variability, exergaming and VR frequently activate central executive and visuomotor networks: practicing increases and decreases prefrontal “cost,” while performance improves with training, and such an efficiency pattern is consistent with findings from training studies and network-level plasticity values [6–9, 12, 14, 32–38]. Programs focusing on balance and complex coordination map onto sensory–vestibular circuits and have been linked to structural and functional remodeling, highlighting activity-specific neuroplasticity [8]. At a lower level, physically active lifestyles seem to modulate microglial dynamics and neuroimmune signaling, providing a potential cellular nexus between cognitively rich physical activity and preserved cognition in ageing [9]. These insights offer one possible explanation for the greater impact of multi-week immersion and interactive programming on executive function and dual-task gait than with exposures within sessions.

Physiological and neurophysiological interpretations partly illustrate this mechanistic picture. Autonomic indices are biased towards increased vagal involvement (increased HRV) across nature exposures [41, 42], while endocrine markers, notably salivary cortisol, are varying and influenced by the characteristics of exercise [43, 44] when sample windows are short or misaligned to diurnal rhythm [16, 26, 45–49]. Higher HRV is linked with more adaptive prefrontal–limbic regulation, a finding that is congruent with the simultaneous increases in affect and executive control that follow enriched sessions [50, 51]. An integration of fNIRS synthesis shows that intensity or concurrent cognitive load influences the hemodynamics of the prefrontal region, whereby the increased levels of oxygenation are associated with increasing task difficulty, a condition that decreases with the additional training as the system becomes cheaper to perform for the same performance, once again a neural efficiency signature [14, 52–55]. Dose and nature-specific design are thus of key importance: one nature walk consistently increases mood in healthy adults, but cognitive change would seem to depend on repeated exposure, additional executive strain, or explicit multi-tasking of physical activity [39, 50]. Virtual “digital shinrin-yoku” may recover some affective and attentional payoffs, but actual experimental data show that the effects are not equal to real nature, and scale according to immersion quality (presence, field of view, latency, interactivity) and scenario structure [41, 56–62]. Similarly, interactive programs that deliberately manipulate task variability, feedback salience, and divided-attention demands are the most likely candidates for achieving large gains in executive control and dual-task gait. Lower-fidelity or one-session exposures can produce transient mood changes that don’t have much long-term cognitive value [5, 32–38].

### Comparison with existing reviews

These trial-level results are directionally consistent with the larger body of meta-analytic and conceptual literature. Experimental findings between green and outdoor exercise indicate small-to-moderate increases in short-term and acute mental outcomes (e.g., positive affect, vitality, restoration), relative to compared indoor or urban exercises, and lower and less consistent differences in physiological improvement [4, 5, 27–44]. The implications of our results expand on this picture by demonstrating that if the dose of exercise is well-matched, the beneficial effects in emotion and intention to participate can still emerge for nature-based conditions, contributing to a plausible motivational route to sustainable motivation for adult exercise continued long-term. Simultaneous evidence from systematic reviews and meta-analyses of VR, exergaming, and similar technology-assisted interventions in older adults and mild cognitive impairment populations highlights performance improvements in executive domains, working memory, and mobility for dual-task conditions. The evidence for these treatments is stronger in cognitively vulnerable groups and among those who receive the intervention in multiple sessions over a period of weeks [4, 5, 27–44]. This pattern aligns with our summarization of more pronounced benefits for exergaming and VR in multi-week dosing and the absence of clear superiority after single sessions.

Indeed, several reviews highlight features of heterogeneity that were apparent across the included studies in their summaries. Differences in immersion fidelity (presence, field of view, interaction latency), task type (e.g., open-skill vs. closed-skill formats), and comparator matching can all significantly impact outcomes, which may help explain why acute endocrine or autonomic effects are less consistent than affective ones [5, 32, 33, 37, 38]. Recent related works focusing on virtual or indoor nature provide similar, albeit partial transfer of restorative gains; however, their effects are sized with more sensory richness and scenario construction, rather than comparable to real outdoor environments [41, 42, 56].

Methodological critiques of this field add to the discussion the requirement for more explicit definitions of exposures, standardized outcomes, and contextual designs for reproducibility and in line with relevant policies [63–67]. These recommendations correlate to the reporting gaps highlighted in our review, for example, their lack of quantification of greenness, noise, and air quality in outdoor arms, as well as an incomplete description of presence or latency in VR conditions. Overall, the findings from previous reviews convey a very similar story: where in the world exercise is happening appears to matter most for mood and restorative experiences, while how immersive or interactive the training is appears more significant for changing executive function and dual-task gait and mobility.

### Strengths and limitations of this review

We made a point of limiting inclusion to randomized trials (parallel and crossover), which facilitates the attribution of causation to the environment or modality as opposed to self-selection. Synthesizing nature-based and immersive, or technology-facilitated, interventions and mapping to shared outcome families (affect and restoration, cognition and dual-task gait, autonomic/endocrine responses, and neural activation) allows for easy comparison between heterogeneous designs. Structured extraction templates, risk-of-bias assessment with RoB 2, and GRADE-based Summary-of-Findings tables further ensure transparent appraisal of certainty and direction of evidence, and our conclusions were evaluated against recent reviews for consistency with the greater field [27–38].

But at the same time, there are key limitations. Considerable heterogeneity of populations, protocols, and endpoint measures prevented us from conducting a quantitative meta-analysis, and many trials were small, time-limited, or had limited endocrine sampling to only one time-point, not necessarily aligned with diurnal patterns. Participants were unable to be blinded, by design, to the environmental or technological context; assessor blinding was uneven, and some crossover trials provided no information about washout or period effects. Reports of exposure fidelity (i.e., greenness, noise, air quality in outdoor arms and presence, latency, or field-of-view in VR conditions) were variable, echoing methodological critiques made on a field level [64]. From a methodological perspective, recent environmental health research has proposed standardized frameworks for quantifying green space exposure, including indicators such as the normalized difference vegetation index, land cover classification, tree canopy coverage, and qualitative environmental metrics [68]. The absence of consistent reporting of such indicators across the included trials limits comparability and underscores the need for improved exposure characterization in future studies. The restriction of the inclusion to English-language, peer-reviewed articles opens up the potential for publication bias that cannot be excluded [27–38, 64], and the lack of individual participant data leaves the following subgroup signals by age, baseline cognition, or functional status to be interpreted with caution from summaries of studies rather than directly modeled.

### Implications and future research

From a public health and service-delivery perspective, the pooled evidence suggests that environmental and modality choices can be used together meaningfully in creating exercise programs for older adults. Nature-based or outdoor walking programs also may be integrated into community and primary-care settings to facilitate affective responses such as perceived restoration and willingness to keep exercise sessions going. These formats are low-cost and widely accessible, making them viable entry points for older adults with poor motivation or low baseline activity. On the contrary, immersive and interactive training based on VR and exergaming might be most useful for cognitive or mobility vulnerabilities (i.e., for EF and dual-task gait) whose functional improvement serves best to decrease fall risk and promotes independence.

More uniform reporting of the exposure parameters — critical for interpretation and replication in future trials — is encouraged. These recommendations address (1) quantifying the environmental or immersive dose equivalently across study arms, (2) providing documentation about immersion characteristics that would underlie cognitive–motor loading across interactive formats, and (3) determining the comparator conditions to sufficiently differentiate between real environmental or technological effects against altered task engagement. By those refinements, future syntheses would enable testing mechanistic hypotheses much more head-on and minimize unexplained heterogeneity.

Although the present review focuses on older adults, affective well-being challenges related to aging contexts extend beyond individuals themselves to include family caregivers and the broader aging ecosystem. Emerging evidence suggests that psychosocial and affective processes among caregivers are closely intertwined with contextual and environmental factors, highlighting the wider relevance of affective outcomes in aging-related health research [69]. Longer and properly powered randomized trials in older and cognitively vulnerable samples will continue to be a priority, particularly if they assess whether enhancements in executive function and dual-task gait and mobility translate into reductions in falls, hospitalization, or loss of independence. Mixed models that integrate community-based outdoor activity with periodic technology-guided sessions may represent a practical approach for scaling up programs in health and social care settings. Intervention implementation studies will also be necessary to identify ways in which these interventions could be incorporated into routine practice (e.g., workforce capacity, supervision requirements, cost–utility) for different subgroups of older adults.

## Conclusions

We find in this review that adding an outdoor or nature-based context to exercise can reliably enhance affective responses, perceived restoration, and willingness to repeat the activity for older adults. Simultaneously, immersive and interactive training formats, such as VR- and exergaming-style exercise that is extended over several weeks, more consistently improve executive performance and dual-task gait than traditional exercise programs. Physiological and endocrine indicators demonstrated more irregularity. Heart rate, heart rate variability, cortisol, and immune indices often reflect the exercise bout itself rather than the environment or technological context. Future trials, then, require greater specificity in measuring environmental and technological exposure—e.g., temperature, air quality, noise, visual field, latency—if we wish to connect exposure to factors with mechanistic pathways.

In terms of public health and service delivery, a pragmatic option is to combine outdoor or nature-based walking and group sessions, which can be provided at very low cost and scaled through community services, with selected technology-assisted sessions for participants who have cognitive or mobility vulnerabilities. Outdoor formats will be able to anchor enjoyment and return intention; interactive VR or exergaming blocks can target executive function and dual-task gait. But larger, longer randomized trials with standardized exposure reporting in older, cognitively vulnerable populations will still be necessary before firm policy recommendations can be made.

## Supplementary Information

Supplementary Material 1.

Supplementary Material 2.

Supplementary Material 3.

Supplementary Material 4.

Supplementary Material 5.

Supplementary Material 6.

Supplementary Material 7.

Supplementary Material 8.

Supplementary Material 9.

Supplementary Material 10.

Supplementary Material 11.

## Data Availability

All search strategies, exclusion lists, RoB rationales, and de-identified extraction tables are supplied in the supplementary files.

## Abbreviations

DLPFC: Dorsolateral Prefrontal Cortex
EEG: Electroencephalography
EXER-TC: Exergaming-based Tai Chi
fNIRS: Functional Near-Infrared Spectroscopy
HR: Heart Rate
HRV: Heart-Rate Variability
IgA: Immunoglobulin A (mucosal immune marker)
MCI: Mild Cognitive Impairment
MoCA: Montreal Cognitive Assessment
PFC: Prefrontal Cortex
RCT: Randomized Controlled Trial
RPE: Rating of Perceived Exertion
RoB 2: Cochrane Risk of Bias 2 Tool for Randomized Trials
VR: Virtual Reality
WM/EF: Working Memory / Executive Function
↑: Improvement/increase in the outcome
↓: Reduction/decrease in the outcome
↔: No evident difference between groups
