# Supplementary material for: Nature-based and technology-assisted exercise for cognitive and mobility outcomes in older adults: a systematic review of randomized trials
Source: BMC Geriatr. 2026 Jan 31;26:282. doi: 10.1186/s12877-026-06978-x (PMC12952035; doi:10.1186/s12877-026-06978-x)
Supplement: Supplementary file 1 — Supplementary Material 1. [file 12877_2026_6978_MOESM1_ESM.docx]

**Manuscript title:**

*Nature-based and Technology-assisted Exercise for Cognitive and Mobility Outcomes in Older Adults: A Systematic Review of Randomized Trials*

**Additional File 1. PRISMA 2020 Checklist**

| **Section and Topic** | **Item** | **Checklist Item** | **Location in Revised Manuscript** |
| --- | --- | --- | --- |
| **TITLE** | 1 | Identify the report as a systematic review | Title page |
| **ABSTRACT** | 2 | Structured summary compliant with PRISMA | Abstract, p. 1–2 |
| **INTRODUCTION** | 3 | Rationale | p. 3 |
|  | 4 | Objectives | p. 4 |
| **METHODS** | 5 | Eligibility criteria | p. 5 |
|  | 6 | Information sources | p. 5 |
|  | 7 | Search strategy | Supplement S1; summary p. 5-6 |
|  | 8 | Selection process | p. 6–7 |
|  | 9 | Data collection process | p. 7–8 |
|  | 10 | Data items | p. 7–8 |
|  | 11 | Study risk of bias assessment | p. 8–9 |
|  | 12 | Effect measures | Not applicable (narrative synthesis only) |
|  | 13a | Synthesis methods: processes used | p. 10 |
|  | 13b | Methods for exploring heterogeneity | Not applicable (no meta-analysis) |
|  | 13c | Synthesis models | Not applicable |
|  | 13d | Sensitivity analyses | Not applicable |
|  | 14 | Reporting bias assessment | Not applicable (no quantitative pooling) |
|  | 15 | Certainty of evidence | Not performed; narrative review only |
| **RESULTS** | 16a | Study selection | p. 7; Figure 1 |
|  | 16b | Reasons for exclusion | Supplement S2 |
|  | 17 | Study characteristics | p. 11–15; Tables 2a, 2b |
|  | 18 | Risk of bias | Supplement S4 |
|  | 19 | Results of individual studies | p. 15–19; Tables 2a, 2b |
|  | 20a | Results of syntheses | p. 19–24 |
|  | 20b | Heterogeneity exploration | Not applicable |
|  | 20c | Sensitivity analysis | Not applicable |
|  | 21 | Reporting biases | p. 25 |
| **DISCUSSION** | 23 | Interpretation | p. 25–29 |
|  | 24 | Limitations of evidence | p. 29–30 |
|  | 25 | Limitations of review processes | p. 29–30 |
|  | 26 | Implications for practice and research | p. 30–31 |
| **OTHER** | 27 | Registration | Not registered (p. 33) |
|  | 28 | Support | p. 33 |
|  | 29 | Competing interests | p. 33 |
|  | 30 | Availability of materials | p. 33 |
