# Supplementary material for: Nature-based and technology-assisted exercise for cognitive and mobility outcomes in older adults: a systematic review of randomized trials
Source: BMC Geriatr. 2026 Jan 31;26:282. doi: 10.1186/s12877-026-06978-x (PMC12952035; doi:10.1186/s12877-026-06978-x)
Supplement: Supplementary file 2 — Supplementary Material 2. [file 12877_2026_6978_MOESM2_ESM.docx]

**Supplementary S1 Full Search Strategies and Exclusions**

**Part A. Full Database Search Strategies (final run: 20–21 Aug 2025; no language filter)**

| **Database** | **Search string (fields/Boolean operators; filters)** | **Records retrieved (n)** |
| --- | --- | --- |
| PubMed | (("green exercise"[Title/Abstract] OR "outdoor exercise"[Title/Abstract] OR forest[Title/Abstract] OR "blue space"[Title/Abstract] OR "nature-based"[Title/Abstract]) OR ("virtual reality"[Title/Abstract] OR exergame [Title/Abstract] OR immersive[Title/Abstract] OR interactive[Title/Abstract])) AND ("executive function"[Title/Abstract] OR "dual-task gait"[Title/Abstract] OR cognition[Title/Abstract] OR "brain activation"[Title/Abstract] OR prefrontal[Title/Abstract] OR affect[Title/Abstract]) AND (randomized[Title/Abstract] OR "randomized controlled trial"[Publication Type] OR crossover[Title/Abstract]) Filters: date range Jan 2010–Aug 2025 | 356 |
| Web of Science (Core Collection) | TS=((green NEAR/3 exercise OR outdoor NEAR/3 exercise OR forest OR "blue space" OR "nature-based") OR ("virtual reality" OR exergam OR immersive OR interactive)) AND TS=("executive function" OR "dual-task gait" OR cognition OR "brain activation" OR prefrontal OR affect) AND TS=(random OR crossover) Timespan: 2010–2025 | 421 |
| Scopus | TITLE-ABS-KEY(((green W/3 exercise OR outdoor W/3 exercise OR forest OR "blue space" OR "nature-based") OR ("virtual reality" OR exergam OR immersive OR interactive)) AND ("executive function" OR "dual-task gait" OR cognition OR "brain activation" OR prefrontal OR affect) AND (random OR crossover)) Date range: 2010–2025 | 612 |
| PsycINFO (EBSCOhost) | (DE "Virtual Reality" OR exergam OR immersive OR interactive OR (green OR outdoor OR forest OR "blue space" OR nature-based) N5 exercise) AND (DE "Executive Function" OR cognition OR "dual-task gait" OR affect OR "brain activation" OR prefrontal) AND (random OR crossover) Date range: 2010–2025 | 143 |
| Total identified |  | 1,532 |

**Part B. Full-text Exclusion Reasons (n = 116)**

| **Reason** | **n** |
| --- | --- |
| Wrong study design | 48 |
| No environmental comparison | 27 |
| Wrong population/setting | 16 |
| Outcomes not relevant | 15 |
| Protocol/abstract only | 6 |
| Duplicate/overlapping data | 4 |

Note: Counts correspond to the PRISMA 2020 flow (Figure 1). Where a study met multiple exclusion reasons, the primary reason was recorded.
