# Supplementary material for: Nature-based and technology-assisted exercise for cognitive and mobility outcomes in older adults: a systematic review of randomized trials
Source: BMC Geriatr. 2026 Jan 31;26:282. doi: 10.1186/s12877-026-06978-x (PMC12952035; doi:10.1186/s12877-026-06978-x)
Supplement: Supplementary file 3 — Supplementary Material 3. [file 12877_2026_6978_MOESM3_ESM.docx]

**Supplement S2. Full-text exclusions (PRISMA-compliant summary)**

Editorial note—availability of itemized exclusions: In accordance with PRISMA 2020, we report full-text exclusion reasons aggregated by standardized categories. The itemized list of excluded full texts (author, year, title/DOI, and primary exclusion reason) will be deposited in an open repository upon acceptance and can be made available to editors and reviewers on request during peer review. This approach avoids duplicative bibliographic disclosure in the manuscript while preserving transparency and reproducibility.

**Part A. Eligibility criteria and screening workflow**

• Designs: randomized controlled trials (parallel) or randomized crossover trials.

• Comparators: (a) nature/outdoor exercise versus indoor/built settings; or (b) immersive/interactive (VR or exergaming) exercise versus conventional training.

• Population: older adults (≥60 years) and MCI; younger-adult studies were collated separately (Supplement S3).

• Outcomes: at least one brain health–related endpoint (affect, cognition, dual-task gait/mobility, neurophysiology, or biomarkers).

• Timeframe and sources: database searches (PubMed, Web of Science, Scopus, PsycINFO/EBSCO) from January 2010 to August 2025, plus citation tracking.

**Part B. Full‑text exclusions by primary reason (n = 116)**

| **Primary reason** | **n** |
| --- | --- |
| Wrong study design (e.g., not randomized/crossover; observational; case report) | 48 |
| No environmental comparison (e.g., only indoor or only outdoor without a comparator) | 27 |
| Wrong population/setting (e.g., pediatric; inpatient; rehabilitation-only) | 16 |
| Outcomes not relevant (no affect/cognition/gait/physiology/biomarker outcomes) | 15 |
| Protocol/abstract only (no full text) | 6 |
| Duplicate/overlapping data (superseded by another paper) | 4 |

**Part C. Transparency and data access**

The detailed, study-level exclusion log (author, year, title/DOI, study design, and primary reason) will be uploaded to an open repository (e.g., OSF) at the time of acceptance with a persistent DOI. During peer review, editors and reviewers may request the log, which we will provide immediately. This maintains fidelity to PRISMA 2020 while keeping the main submission files concise. These materials correspond to PRISMA 2020 Items 16a–16b (study selection and excluded records).
