# Supplementary material for: Nature-based and technology-assisted exercise for cognitive and mobility outcomes in older adults: a systematic review of randomized trials
Source: BMC Geriatr. 2026 Jan 31;26:282. doi: 10.1186/s12877-026-06978-x (PMC12952035; doi:10.1186/s12877-026-06978-x)
Supplement: Supplementary file 4 — Supplementary Material 4. [file 12877_2026_6978_MOESM4_ESM.docx]

**Supplement S3. Data extraction sheet (refs 15–26)**

Variables harmonized by codebook; RoB 2 domain-level summary provided as screening-level judgement.

| **Ref id** | **author year** | **population** | **Design dose** | **context** | **primary outcomes** | **main direction** | **follow up** | **rob2_domain** | **notes** |
| --- | --- | --- | --- | --- | --- | --- | --- | --- | --- |
| [15] | Niedermeier 2017 | Healthy adults (younger) | Randomized crossover; acute ~3 h hike vs treadmill | Nature/outdoor vs indoor | Affect (valence, arousal), fatigue, anxiety | ↑ | Acute | Some concerns | Greater positive affect and lower fatigue/anxiety after mountain hiking vs treadmill |
| [16] | Laezza 2025 | Young men (~25 y) | Randomized crossover; 1 h walk (6 km/h) across settings | Natural vs urban vs indoor | Perceived restoration, affect, intention; HR/HRV | ↑ | Acute | Some concerns | Natural setting improved restoration/affect; HR/HRV trends favorable |
| [17] | Ochiai 2025 | Adults | Parallel RCT; forest vs urban walking | Nature/outdoor vs urban | Mucosal immunity (sIgA), cortisol; mood | ↑ | Acute | Some concerns | Forest walking raised sIgA, reduced cortisol, improved mood |
| [18] | Zukowski 2022 | Older adults (n=60, ~71–72 y) | Parallel RCT; single 30 min session VRTT vs conventional treadmill | VR/exergaming vs conventional | Gait (single/dual-task), cognition (DTE) | ↑ | Acute | Some concerns | Both VR and conventional improved gait/cognition; baseline cognition moderated effects |
| [19] | Ahnesjö 2022 | Healthy older adults | Randomized crossover; fixed RPE across environments | Gradient of nature exposure (three environments) | Heart rate, power output at fixed RPE | → | Acute | Some concerns | Physiologic outputs varied by environment at fixed RPE; not a clinical outcome benefit |
| [20] | Liao 2021 | Frail older adults | Parallel RCT; 12 weeks; 3×60 min/wk; exergaming vs combined exercise | VR/exergaming vs combined exercise | Global cognition; fNIRS (neural efficiency) | ↑ | Post-intervention | Some concerns | Exergaming improved cognition, with signs of increased neural efficiency |
| [21] | Anderson-Hanley 2012 | Older adults | Cluster RCT; ~3 months of cybercycling vs traditional exercise | Exergaming vs conventional | Cognitive function (executive) | ↑ | Post-intervention | Some concerns | Cybercycling outperformed traditional exercise for cognition (same effort) |
| [22] | Liao 2019 | Older adults with MCI | Parallel RCT; 12 weeks; VR-based physical + cognitive training | VR-based training vs conventional | Dual-task gait; executive function | ↑ | Post-intervention | Some concerns | VR group improved dual-task gait; EF gains contributed |
| [23] | Eggenberger 2016 | Older adults | Parallel training; exergame vs balance training | Exergame vs balance | PFC oxygenation during walking (fNIRS); executive function | ↑ | Post-intervention | Some concerns | Training modulated PFC activation; related to EF improvements |
| [24] | Liu 2022 | Older adults with MCI | Parallel RCT; exergaming-based Tai Chi vs traditional Tai Chi | Exergaming-based Tai Chi vs TC | Dual-task gait; executive function | ↑ | Post-intervention | Some concerns | EXER-TC comparable or favorable to TC for dual-task and EF |
| [25] | Zhao 2022 | Older adults (without cognitive impairment) | Parallel training; several weeks exergame vs comparators | Exergame vs aerobic dancing/controls | Working memory; executive inhibition (Stroop) | ↑ | Post-intervention | Some concerns | Long-term exergame improved working memory and inhibitory control benefits |
| [26] | Niedermeier 2017 | Healthy adults (younger) | Randomized trial; acute outdoor vs indoor exercise | Nature/outdoor vs indoor | Cortisol, BP, HRV | → | Acute | Some concerns | Overall endocrine/physiologic differences are limited or mixed |
