# Supplementary material for: Nature-based and technology-assisted exercise for cognitive and mobility outcomes in older adults: a systematic review of randomized trials
Source: BMC Geriatr. 2026 Jan 31;26:282. doi: 10.1186/s12877-026-06978-x (PMC12952035; doi:10.1186/s12877-026-06978-x)
Supplement: Supplementary file 5 — Supplementary Material 5. [file 12877_2026_6978_MOESM5_ESM.docx]

**Supplement S4. RoB 2 item-level judgments with page anchors**

For each trial (Refs 15–26), non‑Low domains include a page anchor (p. x) and a short verbatim justification excerpt.

**Ref 15: RoB 2 domain justifications**

| **Domain** | **Judgment** | **Anchor** | **Verbatim excerpt** |
| --- | --- | --- | --- |
| D1 | Some concerns | p. 1 | randomized crossover trial focusing on |
| D2 | Some concerns | p. 1 | Using a randomized crossover design, 42 healthy participants were randomly exposed to… |
| D4 | Some concerns | p. 3 | post-measurement without a control condition or group. Moreover, most… |

**Ref 16: RoB 2 domain justifications**

| **Domain** | **Judgment** | **Anchor** | **Verbatim excerpt** |
| --- | --- | --- | --- |
| D1 | Some concerns | p. 1 | Evaluating the benefits of green exercise: A randomized controlled trial in… |
| D2 | Some concerns | p. 1 | Using a randomized crossover design, 25 male participants (M =26.3, SD =4.3) completed a 1-h walk at 6 km/h in each setting. Psychological outcomes,… |
| D4 | Some concerns | p. 1 | including perceived restorativeness (PRS), restoration (ROS), emotional states, enjoyment, and behavioral intentions, were assessed with validated questionnaires. |

**Ref 17: RoB 2 domain justifications**

| **Domain** | **Judgment** | **Anchor** | **Verbatim excerpt** |
| --- | --- | --- | --- |
| D1 | Some concerns | p. 1 | OPEN Randomized controlled trial on the… |
| D2 | Some concerns | p. 1 | the need for clear evidence for practical applications remains. We conducted an unblinded randomized… |

**Ref 18: RoB 2 domain justifications**

| **Domain** | **Judgment** | **Anchor** | **Verbatim excerpt** |
| --- | --- | --- | --- |
| D2 | Some concerns | p. 1 | exercise session, participants performed single-task walking, single-task cognitive, and… |

**Ref 19: RoB 2 domain justifications**

| **Domain** | **Judgment** | **Anchor** | **Verbatim excerpt** |
| --- | --- | --- | --- |
| D1 | Some concerns | p. 1 | output among older adults–a randomized… |

**Ref 20: RoB 2 domain justifications**

| **Domain** | **Judgment** | **Anchor** | **Verbatim excerpt** |
| --- | --- | --- | --- |
| D2 | Some concerns | p. 2 | especially in the prefrontal areas, as a compensatory strategy to was a single-blind (assessor), parallel, randomized controlled trial. |
| D4 | Some concerns | p. 2 | especially in the prefrontal areas, as a compensatory strategy to was a single-blind (assessor), parallel, randomized controlled trial. |

**Ref 21: RoB 2 domain justifications**

| **Domain** | **Judgment** | **Anchor** | **Verbatim excerpt** |
| --- | --- | --- | --- |
| D1 | Some concerns | p. 2 | A Cluster Randomized Clinical Trial… |
| D2 | Some concerns | p. 2 | Setting/participants:102 older adults from eight retirement communities enrolled; 79 were… |
| D4 | Some concerns | p. 4 | morestringenttestofthemainhypothesis.37Blindedratingswere Verbal memory(delayed)… |

**Ref 22: RoB 2 domain justifications**

| **Domain** | **Judgment** | **Anchor** | **Verbatim excerpt** |
| --- | --- | --- | --- |
| D2 | Some concerns | p. 2 | Effective intervention in enhancing cognitive function in older adults. This study was a single-blinded (assessor) randomized controlled… |

**Ref 23: RoB 2 domain justifications**

| **Domain** | **Judgment** | **Anchor** | **Verbatim excerpt** |
| --- | --- | --- | --- |
| D1 | Some concerns | p. 1 | were recruited and randomly assigned to either an interactive cognitive-motor video game… |
| D2 | Some concerns | p. 1 | elicits associated changes in cognitive executive functions. Forty-two elderly participants… |
| D4 | Some concerns | p. 3 | balance and stretching group. Participants were blinded to the… |

**Ref 24: RoB 2 domain justifications**

| **Domain** | **Judgment** | **Anchor** | **Verbatim excerpt** |
| --- | --- | --- | --- |
| D2 | Some concerns | p. 2 | single-blind (assessor), parallel, randomized controlled… |

**Ref 25: RoB 2 domain justifications**

| **Domain** | **Judgment** | **Anchor** | **Verbatim excerpt** |
| --- | --- | --- | --- |
| D1 | Some concerns | p. 1 | Fifty-five participants (mean age = 65.4 ± 3.7 years) were randomly assigned to an exergame training (ET) group. |
| D2 | Some concerns | p. 1 | Fifty-five participants (mean age = 65.4 ± 3.7 years) were randomly assigned to exergame training (ET). |
| D3 | Some concerns |  | (no direct phrase matched; see Methods/Results) |
| D4 | Some concerns |  | (no direct phrase matched; see Methods/Results) |
| D5 | Some concerns | p. 1 | During 12 weeks, outcome measures for cognitive function included working memory. |

**Ref 26: RoB 2 domain justifications**

| **Domain** | **Judgment** | **Anchor** | **Verbatim excerpt** |
| --- | --- | --- | --- |
| D1 | Some concerns | p. 1 | A Randomized Crossover Trial on Acute… |
| D2 | Some concerns | p. 1 | physiological responses. Using a randomized crossover design, 42 healthy participants were… |
| D4 | Some concerns | p. 3 | measurements, the three-hour intervention started (outdoor mountain hiking, indoor treadmill… |

**Domain Notes**

D1: Bias arising from the randomization process

D2: Bias due to deviations from intended interventions

D3: Bias due to missing outcome data

D4: Bias in measurement of the outcome

D5: Bias in the selection of the reported result
