# Supplementary material for: Nature-based and technology-assisted exercise for cognitive and mobility outcomes in older adults: a systematic review of randomized trials
Source: BMC Geriatr. 2026 Jan 31;26:282. doi: 10.1186/s12877-026-06978-x (PMC12952035; doi:10.1186/s12877-026-06978-x)
Supplement: Supplementary file 6 — Supplementary Material 6. [file 12877_2026_6978_MOESM6_ESM.docx]

**Supplement S5. Summary of Findings (GRADE Table)**

Nature-based and Technology-assisted Exercise for Cognitive and Mobility Outcomes in Older Adults and Mild Cognitive Impairment (MCI)

| **Outcome domain** | **No. of trials (participants)** | **Comparison (intervention vs control)** | **Effect direction** | **Certainty of evidence (GRADE)** | **Summary of findings and interpretation** |
| --- | --- | --- | --- | --- | --- |
| Executive and global cognitive function | 6 RCTs (n ≈ 360) | Exergaming / VR vs conventional or combined exercise | ↑ improvement | ⬤⬤◯◯ Low | Moderate improvement in global cognition and executive function; consistent trends across 4 trials (Liao 2019, 2021; Liu 2022; Anderson-Hanley 2012). Limitations include small samples and a lack of blinding. |
| Dual-task gait and motor–cognitive interaction | 4 RCTs (n ≈ 240) | VR/exergaming vs traditional training | ↑ improvement | ⬤⬤◯◯ Low | Significant gains in dual-task cost, gait speed, and postural control. Benefits are most pronounced in MCI subgroups. Heterogeneity is moderate due to different devices. |
| Affect and well-being | 3 crossover trials (n ≈ 100) | Outdoor/nature exercise vs indoor/urban | ↑ improvement | ⬤⬤⬤◯ Moderate | Outdoor exposure improved positive affect and perceived restoration immediately post-exercise (Niedermeier, 2017; Laezza, 2025). Physiological strain matched controls, suggesting psychological mediation. |
| Physiological stress and neuroendocrine markers | 2 crossover RCTs (n ≈ 70) | Forest/nature walking vs urban or indoor | → no clear difference | ⬤⬤◯◯ Low | Minor or inconsistent changes in cortisol, HRV, and BP; possible ceiling effect in healthy samples. |
| Prefrontal activation and neural efficiency (fNIRS/fMRI) | 3 RCTs (n ≈ 150) | Exergaming / VR vs conventional | ↑ improvement | ⬤⬤⬤◯ Moderate | VR and exergaming elicited reduced PFC oxygenation during dual-task walking, indicating improved neural efficiency (Eggenberger 2016; Liao 2021). |
| Adherence and feasibility | 5 RCTs (n ≈ 320) | All modalities | ↑ adherence | ⬤⬤⬤◯ Moderate | High adherence (≥85%) across both outdoor and VR programs. Minor adverse events (muscle soreness, visual fatigue) reported in <5%. |

**Interpretation and confidence in evidence**

Overall, the certainty of evidence was moderate to low, primarily due to small samples, short follow-up durations, and limited blinding feasibility. Nature-based interventions demonstrate consistent affective and motivational benefits, with possible cognitive spill-over via mood regulation and environmental engagement. VR and exergaming interventions show potential for enhancing executive and dual-task functions, supported by objective neural markers. Physiological evidence remains inconclusive but supports feasibility and safety in older adults and MCI. Further large-scale, longer-term RCTs with standardized cognitive outcomes and neuroimaging endpoints are warranted.

**GRADE symbols**

| **Symbol** | **Level** | **Interpretation** |
| --- | --- | --- |
| ⬤⬤⬤⬤ | High | Further research is unlikely to change confidence in the estimate of effect. |
| ⬤⬤⬤◯ | Moderate | Further research is likely to have a significant impact and may change the estimate. |
| ⬤⬤◯◯ | Low | Further research is very likely to change the estimate of effect. |
| ⬤◯◯◯ | Very low | Any estimate of effect is very uncertain. |
