# Supplementary material for: Nature-based and technology-assisted exercise for cognitive and mobility outcomes in older adults: a systematic review of randomized trials
Source: BMC Geriatr. 2026 Jan 31;26:282. doi: 10.1186/s12877-026-06978-x (PMC12952035; doi:10.1186/s12877-026-06978-x)
Supplement: Supplementary file 7 — Supplementary Material 7. [file 12877_2026_6978_MOESM7_ESM.docx]

**Supplement S6. Younger-Adult and Middle-Aged Randomized Trials (not included in primary synthesis/** **for completeness)**

These studies enrolled non-older adult samples and are presented here to keep the main text focused on older adults and MCI.

| **Study**  **[Ref]** | **Country** | **Sample Size (Intervention / Control)** | **Age (mean ± SD)** | **Population** | **Intervention** | **Comparator** | **Key Outcomes** | **Note** |
| --- | --- | --- | --- | --- | --- | --- | --- | --- |
| Niedermeier 2017 [15] | Austria | 20 / 20 | 32.0 ± NR | Healthy young adults | Outdoor green exercise vs indoor exercise | Indoor gym-based exercise | Mood, affect, attention | Not included in primary synthesis |
| Niedermeier 2017 (second trial) [26] | Austria | 22 / 22 | 32.2 ± NR | Healthy young adults | Outdoor vs indoor cycling | Indoor cycling | Affect, cognitive processing | Supplement only |
| Laezza 2025 [16] | Italy | 30 / 30 | 26.3 ± NR | Young adults | Immersive VR walking | Non-immersive VR or standard walking | Executive function, spatial navigation | Younger-adult VR trial |
| Ochiai 2025 [17] | Japan | 18 / 18 | 53.05 ± NR | Middle-aged adults | VR-based balance and mobility training | Conventional balance training | Balance indices, gait control | Middle-aged group; not included in primary synthesis |

*These younger-adult and middle-aged trials were retained for transparency and methodological completeness but were not included in the primary tables, synthesis, or conclusions of this review.*
