# Supplementary material for: Nature-based and technology-assisted exercise for cognitive and mobility outcomes in older adults: a systematic review of randomized trials
Source: BMC Geriatr. 2026 Jan 31;26:282. doi: 10.1186/s12877-026-06978-x (PMC12952035; doi:10.1186/s12877-026-06978-x)
Supplement: Supplementary file 8 — Supplementary Material 8. [file 12877_2026_6978_MOESM8_ESM.docx]

**Supplement** **S7. Exposure Fidelity Assessment Matrix for Included Randomized Trials**

| **Study** | **Exposure type** | **Key exposure descriptors reported** | **Fidelity rating** | **Rationale** |
| --- | --- | --- | --- | --- |
| Niedermeier et al., 2017 [15] | Nature-based (outdoor hiking) | Route type, altitude, duration, terrain | Moderate | Setting described but no quantitative greenness or canopy metrics |
| Laezza et al., 2025 [16] | Nature-based (green vs built) | Environmental categorization, restorative properties | Moderate | Perceived restoration assessed, but no objective greenness indices |
| Ochiai et al., 2025 [17] | Nature-based (forest walking) | Forest setting, walking protocol | Moderate | Natural environment specified, NDVI/canopy not quantified |
| Zukowski et al., 2022 [18] | Technology-assisted (VR treadmill) | VR treadmill paradigm, task description | Moderate | Device described; no field of view or latency reported |
| Ahnesjö et al., 2022 [19] | Nature-based vs indoor | Environment type, exercise dose | Low–Moderate | Environment labeled but limited contextual detail |
| Liao et al., 2021 [20] | Exergaming | Platform, cognitive–motor task design | High | Detailed task structure and progression described |
| Anderson-Hanley et al., 2012 [21] | Exergaming (cybercycle) | Device type, interactive cycling task | Moderate | Device named; limited technical specifications |
| Liao et al., 2019 [22] | VR-based physical–cognitive | VR task paradigm, cognitive–motor integration | Moderate | Training described; immersion parameters not reported |
| Eggenberger et al., 2016 [23] | Exergame + balance | Balance tasks, progression, fNIRS integration | High | Clear exposure description enabling reproducibility |
| Liu et al., 2022 [24] | Exergaming-based Tai Chi | Task structure, Tai Chi adaptation | Moderate | Task described; immersive characteristics limited |
| Zhao et al., 2022 [25] | Exergame training | Cognitive task integration, session structure | Moderate | Intervention logic clear; device details limited |
| Niedermeier et al., 2017 [26] | Outdoor vs indoor exercise | Environment contrast, physiological measures | Low–Moderate | Exposure contrast clear, contextual detail limited |

**Interpretation note**

Fidelity ratings represent the transparency and reproducibility of exposure reporting rather than exposure “dose” or intervention quality. Across trials, moderate fidelity was most common, reflecting a general lack of standardized environmental or immersive metrics in the literature.
