# Supplementary material for: Nature-based and technology-assisted exercise for cognitive and mobility outcomes in older adults: a systematic review of randomized trials
Source: BMC Geriatr. 2026 Jan 31;26:282. doi: 10.1186/s12877-026-06978-x (PMC12952035; doi:10.1186/s12877-026-06978-x)
Supplement: Supplementary file 9 — Supplementary Material 9. [file 12877_2026_6978_MOESM9_ESM.docx]

**Supplement S8. Standardized Exposure Reporting Checklist for Nature-Based and Technology-Assisted Exercise Trials**

**Purpose**

This checklist is intended to improve transparency, reproducibility, and cross-study comparability in randomized trials that treat exercise environment or immersion as a primary exposure. Items are organized by exposure category and are recommended for reporting alongside standard exercise prescription variables (frequency, intensity, time, and type).

**Scope**

The checklist applies to trials examining nature-based (outdoor or green/blue space) exercise and technology-assisted (virtual reality or exergaming) exercise in older adults and related populations.

**A. Core Reporting Items (Applicable to All Trials)**

| **Item** | **Description** |
| --- | --- |
| Study design | Parallel, crossover, cluster randomized; acute vs training |
| Population | Age range, mean age, cognitive status (e.g., healthy, frail, MCI) |
| Exercise prescription | Mode, frequency, duration, intensity target |
| Comparator description | Indoor, usual care, conventional exercise; level of matching |
| Session supervision | Supervised, partially supervised, unsupervised |
| Adherence and compliance | Attendance, completion rate, deviations |
| Adverse events | Falls, cybersickness, discomfort, withdrawals |

**B. Nature-Based Exercise Exposure Reporting**

| **Item** | **Recommended description** |
| --- | --- |
| Setting type | Park, forest, urban green space, blue space, mixed |
| Route characteristics | Distance, elevation, terrain (flat, uneven, trail) |
| Greenness indicators | NDVI, tree canopy cover, or validated proxy if available |
| Environmental context | Urban vs rural; proximity to traffic or buildings |
| Sensory context | Visual dominance, noise levels (qualitative or quantitative), air quality if available |
| Weather conditions | Temperature, precipitation, season |
| Session timing | Time of day, daylight exposure |
| Group vs individual | Solo, group-based, guided |

**Minimum reporting standard:** clear description of setting type and route characteristics.

**Enhanced reporting:** inclusion of objective greenness or environmental metrics.

**C. Technology-Assisted Exercise Exposure Reporting (VR / Exergaming)**

| **Item** | **Recommended description** |
| --- | --- |
| Platform type | Exergaming console, semi-immersive VR, fully immersive VR |
| Device model | Manufacturer and model name |
| Display characteristics | Screen vs head-mounted display |
| Field of view | Horizontal and vertical degrees, if available |
| Refresh rate/latency | Frame rate, motion-to-photon latency |
| Interaction mode | Controller, body tracking, treadmill, balance board |
| Task structure | Cognitive demands, feedback type, progression |
| Immersion indicators | Presence questionnaire or proxy |
| Cybersickness assessment | Symptoms monitored and reported |

**Minimum reporting standard:** platform type, task description, and interaction mode.

**Enhanced reporting:** device-level specifications and immersion metrics.

**D. Exposure Fidelity and Reproducibility**

| **Item** | **Description** |
| --- | --- |
| Exposure rationale | Why this environment or technology was selected |
| Fidelity monitoring | Checks to ensure exposure consistency across sessions |
| Co-interventions | Concurrent cognitive tasks or sensory manipulation |
| Adaptation over time | Progression or variation of exposure |
| Replicability | Sufficient detail to reproduce exposure in another setting |

**E. Recommended Reporting Summary**

Authors are encouraged to include a brief **Exposure Reporting Summary** table outlining key environmental or immersive features alongside outcome reporting. When objective metrics are unavailable, qualitative descriptors and rationale should be explicitly stated.

**Interpretation note**

This checklist does not prescribe specific technologies or environmental metrics but aims to standardize reporting of exposure characteristics that are critical when environment or immersion is treated as an active intervention component.
