# Supplementary material for: Nature-based and technology-assisted exercise for cognitive and mobility outcomes in older adults: a systematic review of randomized trials
Source: BMC Geriatr. 2026 Jan 31;26:282. doi: 10.1186/s12877-026-06978-x (PMC12952035; doi:10.1186/s12877-026-06978-x)
Supplement: Supplementary file 10 — Supplementary Material 10. [file 12877_2026_6978_MOESM10_ESM.docx]

**Supplement** **S9. Operational Definitions of Participant Characteristics**

| **Study** | **Population label used in review** | **Diagnostic or screening criteria reported** | **Cutoff or description** |
| --- | --- | --- | --- |
| Niedermeier et al., 2017 [15] | Healthy older adults | Self-reported health status | No frailty or cognitive impairment criteria reported |
| Laezza et al., 2025 [16] | Healthy older adults | Community-dwelling older adults | No frailty or cognitive screening reported |
| Ochiai et al., 2025 [17] | Healthy older adults | Community-based sample | No formal frailty or cognitive diagnosis reported |
| Zukowski et al., 2022 [18] | Healthy older adults | Inclusion based on age and mobility | Cognitive impairment excluded |
| Anderson-Hanley et al., 2012 [21] | Cognitively vulnerable older adults | Mini-Mental State Examination (MMSE) | MMSE < 28 |
| Eggenberger et al., 2016 [23] | Older adults with mild cognitive impairment | Petersen criteria | Clinical diagnosis of MCI |
| Liao et al., 2019 [22] | Older adults with mild cognitive impairment | Montreal Cognitive Assessment (MoCA) | MoCA < 26 |
| Liao et al., 2021 [20] | Cognitively vulnerable older adults | Montreal Cognitive Assessment (MoCA) | MoCA 22–26 |
| Liu et al., 2022 [24] | Frail older adults | Fried frailty phenotype | ≥3 frailty criteria |
| Zhao et al., 2022 [25] | Frail older adults | Fried frailty phenotype | ≥3 frailty criteria |
| Ahnesjö et al., 2022 [19] | Older adults | Clinical screening | Frailty and cognitive impairment excluded |
| Niedermeier et al., 2017 [26] | Older adults | Community-dwelling sample | No formal frailty or cognitive classification |

**Interpretation note**

Population descriptors used throughout the review reflect the criteria explicitly reported in each original trial. Considerable heterogeneity exists in frailty and cognitive classification approaches, including use of validated phenotypes, screening instruments, or clinical diagnosis. These differences were not harmonized across studies and should be considered when interpreting subgroup patterns.
