# Supplementary material for: Nature-based and technology-assisted exercise for cognitive and mobility outcomes in older adults: a systematic review of randomized trials
Source: BMC Geriatr. 2026 Jan 31;26:282. doi: 10.1186/s12877-026-06978-x (PMC12952035; doi:10.1186/s12877-026-06978-x)
Supplement: Supplementary file 11 — Supplementary Material 11. [file 12877_2026_6978_MOESM11_ESM.docx]

**Supplement** **S10. Cognitive Tasks Used in Dual-Task Gait Paradigms**

| **Study** | **Gait task** | **Cognitive task performed during gait** | **Cognitive domain** |
| --- | --- | --- | --- |
| Eggenberger et al., 2016 [23] | Overground walking | Serial subtraction (counting backwards by 7s) | Working memory, attention |
| Liao et al., 2019 [22] | Treadmill walking | Verbal fluency (category naming) | Executive function, language |
| Liao et al., 2021 [20] | Overground walking | Stroop-like color–word interference task | Inhibitory control, executive function |
| Liu et al., 2022 [24] | Overground walking | Serial subtraction (counting backwards by 3s) | Attention, working memory |
| Zhao et al., 2022 [25] | Overground walking | Verbal fluency (letter naming) | Executive function |
| Anderson-Hanley et al., 2012 [21] | Treadmill walking | Verbal arithmetic task | Attention, working memory |

**Interpretation note**

Considerable heterogeneity exists in the cognitive tasks paired with gait across studies, spanning domains of attention, working memory, inhibitory control, and executive function. These differences should be considered when interpreting dual-task gait outcomes and comparing effect patterns across trials.
